# Comparison of automated irrigation systems using an in vitro ureteroscopy model

**DOI:** 10.1590/S1677-5538.IBJU.2019.0230

**Published:** 2020-02-20

**Authors:** Donald Fedrigon, Luay Alshara, Manoj Monga

**Affiliations:** 1 Cleveland Clinic Glickman Kidney St Urological Institute ClevelandOH USA Cleveland Clinic, Glickman Kidney St Urological Institute, Cleveland, OH, USA

**Keywords:** Research, Technology, Ureteroscopy

## Abstract

**Introduction:**

Two automated irrigation systems have been released for use during endoscopic procedures such as ureteroscopy: the Cogentix RocaFlow® (CRF) and Thermedx FluidSmart® (TFS). Accurate pressure control using automated systems may help providers maintain irrigation pressures within a safe range while also providing clear visualization. Our objective was to directly compare these systems based on their pressure accuracy, pressure-flow relationships, and fluid heating capabilities in order to help providers better utilize the temperature and pressure settings of each system.

**Materials and Methods:**

An in vitro ureteroscopy model was used for testing, consisting of a short semirigid ureteroscope (6/7, 5F, 31cm Wolf 425612) connected to a continuous digital pressure transducer (Meriam m1550). Each system pressure output and flow-rate, via 100mL beaker filling time, was measured using multiple trials at pressure settings between 30 and 300mmHg. Output fluid temperature was monitored using a digital thermometer (Omega DP25-TH).

**Results:**

The pressure output of both systems exceeded the desired setting across the entire tested range, a difference of 15.7±2.4mmHg for the TFS compared to 5.2±1.5mmHg for the CRF (p <0.0001). Related to this finding, the TFS also had slightly higher flow rates across all trials (7±2mL/min). Temperature testing revealed a similar maximum temperature of 34.0°C with both systems, however, the TFS peaked after only 8 minutes and started to plateau as early as 4-5 minutes into the test, while the CRF took over 18 minutes to reach a similar peak.

**Conclusions:**

Our in vitro ureteroscopy testing found that the CRF system had better pressure accuracy than the TFS system but with noticeably slower fluid heating capabilities. Each system provided steady irrigation at safe pressures within their expected operating parameters with small differences in performance that should not limit their ability to provide steady irrigation at safe pressures.

## INTRODUCTION

Endoscopic urological procedures require adequate irrigation to ensure clear visualization and efficient stone clearance throughout procedures such as ureteroscopy. Ureteroscopes often require pressurized irrigation since they utilize a small, shared working and irrigation channel, which increases resistance. Adequate irrigation is important for dilation of the ureter and pelvicalyceal system, enhancing instrument passage and visibility.

While pressurized irrigation during endoscopic procedures is often necessary for clear visualization and efficient stone clearance, its use can also lead to elevated renal pelvis pressures (RPP). This elevation in pressure may cause retrograde flow of fluid, bacteria, and/or endotoxins from the urinary collecting system into the systemic venous circulation, referred to as pyelovenous backflow (1). Therefore, accurate pressure control using automated systems helps providers maintain irrigation pressures within a safe range throughout the procedure.

Various techniques have been used to enhance irrigation, including gravity drainage, pressurized irrigation bags, and handheld or foot activated bulb or syringe-based systems (2). More recently, two automated systems have entered the market to provide digital temperature and pressure monitoring, the Thermedx FluidSmart (TFS) ® and the Cogentix RocaFlow (CRF) ®. Both systems provide continuous irrigation with pressure control and fluid warming.

The Thermedx FluidSmart (TFS) ® system can provide irrigation pressures between 30 and 300mmHg via a rollerball pump. The Cogentix RocaFlow (CRF) ® system can provide pressures up to 735mmHg (1000cmH_2_O) via two chambers pressurized with compressed medical air which house the saline bags. Each system's pressure settings is operated via touchscreen and has procedure and specialty specific profiles including transurethral resections, ureteroscopy, and percutaneous nephrolithotomy. The fluid warming system employed by each system is slightly different. The TFS tubing includes a fluid distribution cartridge that slots into a heating unit which warms exiting fluid, which can be set by the user to a maximum of 40°C. The CRF heats each chamber's respective saline bag, which is preset to 38±2°C. Both systems have the option to provide suction fluid return as well. The TFS also has a continuous monitoring system to display the current temperature and flow rate as well as to record the fluid usage volume, total fluid deficit, and average temperature.

A previous in vitro analysis of the TFS system was performed at our institution using a rigid ureteroscopy model to characterize the rate of temperature change, pressure accuracy, and the precision of the continuous pressure monitoring (3). This analysis demonstrated that the TFS system overestimated the temperature and flow rate while underestimating the pressure supplied, however, these discrepancies were not significant enough to limit functionality or safety (3). As far as we are aware, no similar in vitro or in vivo comparisons using the Thermedx FluidSmart® or the Cogentix RocaFlow® have been conducted since this publication. The objective of this study was to directly compare both automated systems based on irrigating pressure accuracy, pressure-flow relationships, and fluid heating efficiency in order to help providers better utilize the temperature and pressure settings of each system.

## MATERIALS AND METHODS

**Figure 1:**
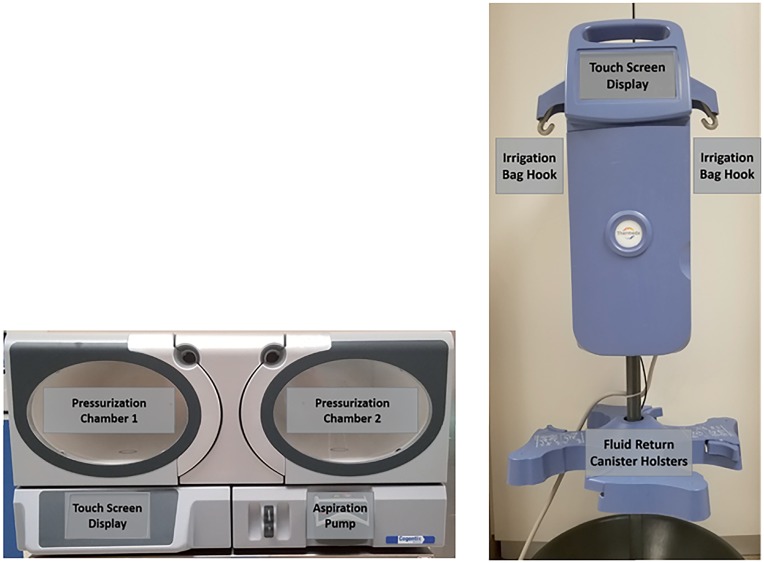
Left: Cogentix RocaFlow (CRF), Right: Thermedx FluidSmart (TFS).

Pressure and temperature measurements were performed using a continuous digital pressure transmitter (Meriam m1550) and a continuous read digital thermometer (Omega DP25-TH), respectively. Each system was tested using an in vitro ureteroscopy model with a short semirigid ureteroscope (6/7, 5F, 31cm Wolf 425612). For all tests each system was operated combined with the appropriate tubing set and room-temperature 3L saline bags. Tests for pressure and flow rate were repeated at fourteen different pressure settings across the urology relevant range of 30 to 300mmHg. Due to the preset increments in the CRF system increasing by only 6-7mmHg, the tests for pressures for 95, 140, 200, 245, and 260mmHg were run at 97, 142, 202, 247, and 262mmHg respectively. All calculations were done using the exact pressure used for each system but are displayed as equal in the figures for easier comparison (Figure-1).

### Pressure Accuracy

**Figure 2:**
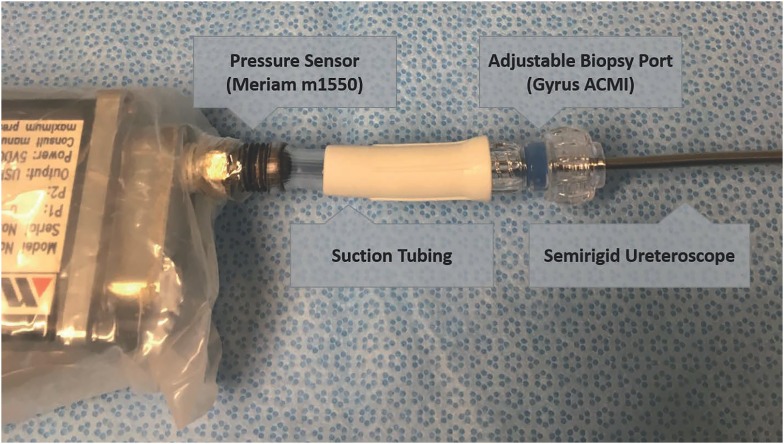
Pressure testing setup using fluid filled section of tubing attached to the semirigid ureteroscope at one end and the pressure transducer at the other.

Pressure tests were conducted with a one-inch section of suction tubing connecting the pressure sensor and an adjustable biopsy port (Gyrus ACMI), through which the tip of the scope was inserted before tightening to ensure a watertight seal. Both connections were secured with pipe-fitters tape to prevent leakage (Figure-2). Each irrigation system was attached to the scope directly without any intervening stopcocks or Luer locks using the provided flexible tubing section for the Rocaflow and the cut end of the Thermedx tubing.

For the TFS, the Thermedx Disposable Ureteroscopy Tubing Set includes a mechanical pressure release valve which is not present in the CRF system. The CRF can be used with both the TURP Tubing Set and Traxerflow Ureteroscopy Tubing Set, with the latter including a hand pump segment with an anti-return valve. In order to maintain consistency neither the TFS pressure release valve or the CRF hand pump sections were used. The pressure sensor and irrigation systems remained level with each other to prevent any gravity influence for both pressure and flow rate tests. Trials were repeated multiple times for each pressure setting and the average used for all calculations.

### Flow Rate

Flow rates through the ureteroscope were calculated by recording the time to fill a 100mL beaker at a range of pressure settings. Trials were repeated multiple times for each pressure setting and the average time reported.

### Fluid Temperature

The initial fluid temperature, ambient room temperature, and time to maximum temperature were recorded with each system. Ambient room temperature for all tests was between 19.0-20.0°C and starting fluid temperature was 20.2-20.6°C unless otherwise noted. Both systems were started from their off state without warmup time and trials were spaced apart to allow each system to cool down between runs. Irrigation fluid temperature was measured with the probe 1cm from the scope tip using standardized flow rates based on the above measurements to provide an equal flow rate for both systems. The TFS system was set to its maximum temperature setting of 40°C for all tests.

### Statistics

Statistical analysis was performed with two-tailed independent t-tests for continuous means. For all tests p-values <0.05 were considered statistically significant. Statistical analysis was performed using R Statistical Software (R Foundation for Statistical Computing, Vienna, Austria).

## RESULTS

### Pressure Accuracy

**Figure 3:**
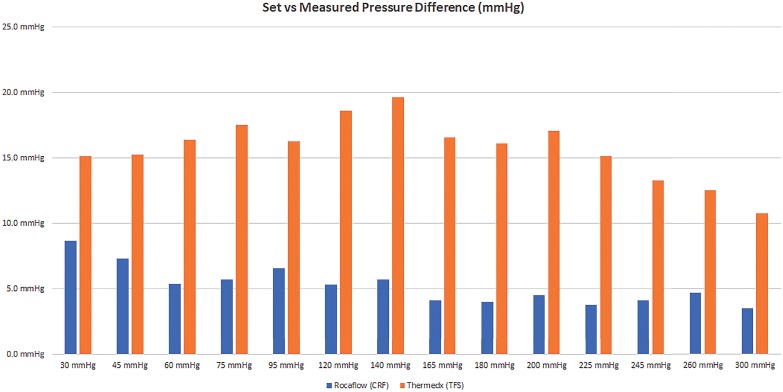
Difference between observed fluid pressure output and set pressure at each pressure increment. Multiple trials repeated at each pressure setting with average displayed.

For all measured pressures the TFS and CRF systems provided a pressure output above the setpoint set on each system. The TFS demonstrated a pressure difference of 15.7±2.4mmHg compared to 5.2±1.5mmHg for the CRF (mean±SD), this difference in accuracy was statistically significant (p <0.0001, Figure-3). There was no observed trend in pressure output accuracy at the range of pressures tested between 30 and 300mmHg.

### Flow Rate

**Figure 4:**
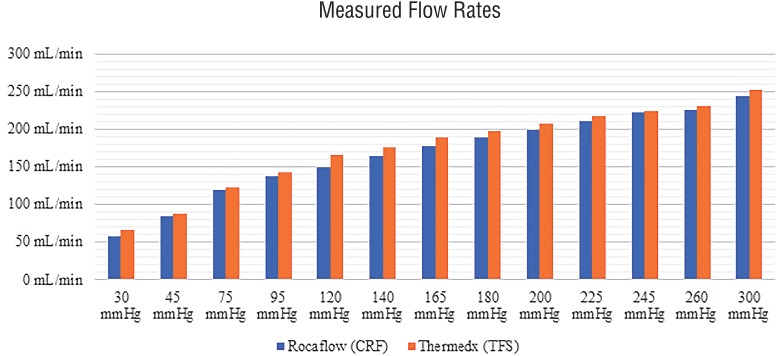
Observed flow rates as measured by time to fill 100 mL beaker, average time of multiple trials displayed.

The measured flow rates at each pressure setting for each system are displayed in Figure-4. We observed a slightly higher flow rate from the TFS system, on average 7±2mL/min higher than the CRF systems outputs.

### Fluid Temperature

**Figure 5:**
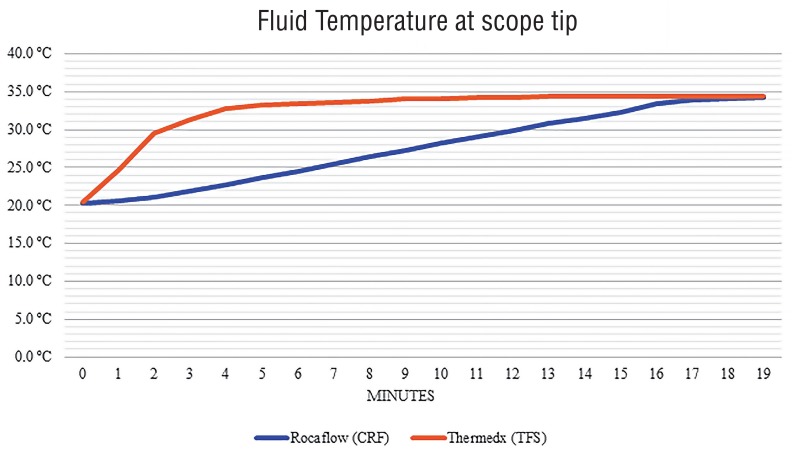
Temperature of irrigation fluid as measured 1 cm from the scope tip.

To test the consistency of each system heating mechanism, each system was measured at 136, 164, and 200mL/min flow rates, which did not reveal any consistent differences in rate of temperature increase or maximum temperature. Therefore, the average temperature trend starting from room temperature was reported to provide data generalizable to a variety of usage patterns (Figure-5). The TFS fluid output was heated to above 34.0°C, close to the maximum of each system after only 8 minutes and started to plateau as early as 4-5 minutes into the test. The CRF system took over 18 minutes to reach the same temperature of 34.0°C and demonstrated a more gradual temperature slope.

**Figure 6:**
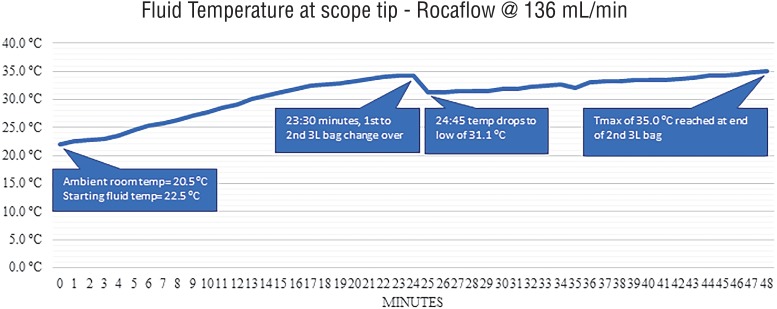
Temperature at scope tip during full length test of two 3L saline bags.

Additionally, we conducted a longer test of the CRF system using two 3L saline bags from room temperature to simulate real-world use when depleting a single bag before switching to the second chamber. During this test the fluid reached a maximum of 35.0°C at the tail-end of the second bag with a drop from 34.2 to 31.1°C following the transition between the bags. The average temperature throughout the 48-minute test was 30.8°C (Figure-6).

## DISCUSSION

Both the TFS and CRF are automated irrigation systems with pressure and temperature control for the purpose of providing steady irrigation during ureteroscopy. In our previous study evaluating only the Thermedx system, we found that the TFS monitoring system underestimated pressures and overestimated both flow rates and temperatures delivered through the endoscope (3). In this study, we retested both the TFS with the addition of the CRF system, with a focus on pressure accuracy.

### Pressure Accuracy

During our testing both systems overestimated the pressure output. However, of the two automated systems the CRF showed better pressure accuracy than the TFS, based on absolute difference between the set and measured pressures.

Gravity based systems and pressurized bag sleeves may cause fluctuations in the output pressure since they cannot account for changes in resistance within the working channel or at the scope tip. The new automated systems aim to address this by monitoring the fluid pressure and adjusting their output to maintain a steady output pressure. These improvements aim to improve visualization, provide a larger working space, and allowing easier progression of instruments in the hope of reducing operative times and improving stone free rates. Lechevallier et al. showed significant reduction in the mean operative time among patients randomized to an automated pressurized irrigation system compared to standard pressurized irrigation, 32% with the rigid instrument and 53% with the flexible instrument, which the authors attributed to the improved working space and visibility (4).

Pressure accuracy is also important for preventing the retrograde flow of fluid, bacteria, and/or endotoxins from the urinary collecting system into the systemic venous circulation, referred to as pyelovenous or pyelolymphatic backflow, commonly believed to occur at pressures greater than 30mmHg. Previous literature estimates that the fluid absorption from pressures in excess of this threshold during ureteroscopy is fairly limited compared to percutaneous nephrolithotomy (1, 5). Since both automated irrigation systems are intended for use during both procedures, our pressure accuracy analysis is likely relevant to both procedures. These systems may provide better control to minimize any unwanted increase in pressures above the threshold without compromising visibility and operative time.

### Flow Rate

We observed slightly higher flow rates for the TFS system across all tested pressures, which may be a by-product of the higher-pressure output of this system. Subjectively, we also observed during our flow testing that the CRF system demonstrated a slightly less variable flow, similar to what would be expected from passive gravity irrigation.

Most irrigation systems in use today utilize either passive gravity-driven flow or active irrigation provided by hand or foot-operated pumps. For pump operated systems, flow rate fluctuations may cause more erratic movement of stone fragments. Gravity based systems on the other hand, may exert less force than active irrigation systems (6). Minimizing stone migration during ureteroscopy by providing a more tempered, steadier flow while also maintaining adequate force via an automated system would therefore be expected to correlate with reduced operative times.

Proper irrigation is important not only for minimizing stone migration and renal pelvis pressures but also for proper temperature control during laser lithotripsy. A recent in vitro analysis by Wollin et al. found that while adequate irrigation can maintain stable temperatures across a range of laser settings, decreasing irrigation rates can result in potentially dangerous temperature elevations even with low power laser use (7). An ex vivo model used by Molina et al. observed that irrigation decreased external ureteral temperature elevations during laser lithotripsy (8).

### Fluid Temperature

While both systems eventually reached a similar maximum temperature of 35°C, still below the advertised temperature set point for both systems, the CRF system took much longer to reach this maximum. This appears to be a reflection of the different approaches to fluid warming used by each system. The CRF heats the entire bag within the chamber and therefore showed a steady rise throughout the test, with the maximum only being achieved when a small amount of fluid remained in the bag to be heated. The additional time to heat more fluid was also observed in our longer trial showing a temperature dip when switching to a full bag that had been heating in the second chamber. The TFS system meanwhile, heats only the small amount of fluid exiting the machine and therefore reached its maximum temperature in a much shorter period as the internal heating element warms to the proper temperature.

The usage of room temperature instead of warmed irrigation fluids during some endourological procedures such as percutaneous nephrolithotomy has been associated with significant decreases in body temperature as well as longer anesthesia recovery times (9). Additionally, mild perioperative hypothermia has been associated with adverse events such as increased blood loss, weakened immune responses, and discharge times (10). Therefore, having an efficient heating component during these high volume procedures may have a noticeable impact on patient outcomes.

This study is limited by its in vitro nature since the irrigation systems characteristics and measurements may be affected by the physiological properties of a real kidney and urological system. Additionally, in order to maintain consistency between the two irrigation systems tubing we excluded some elements that could affect pressures such as release valves and hand-pump segments. Future in vivo studies may help confirm their clinical applicability and cost in order to help providers better understand the operating characteristics during the use of these systems.

## CONCLUSIONS

When comparing automated irrigation systems using an in vitro ureteroscopy model the Cogentix RocaFlow (CRF) ® system demonstrated more accurate pressure output compared to the Thermedx FluidSmart (TFS) ® system. While both systems reached a similar peak temperature output, the CRF system showed noticeably slower heating capabilities. Despite these differences in operating characteristics both systems performed within their expected parameters, with small variations that should not limit their ability to provide steady irrigation at safe pressures.

## ABBREVIATIONS

CRF: Cogentix RocaFlow
mmHg: Millimeter of mercury
SD: Standard deviation
TFS: Thermedx FluidSmart
